# The role of the FKBP51–Hsp90 complex in Alzheimer’s disease: An emerging new drug target

**DOI:** 10.1016/j.cstres.2024.11.006

**Published:** 2024-11-29

**Authors:** Xavier Jeanne, Zsolt Török, László Vigh, Chrisostomos Prodromou

**Affiliations:** 1 Biochemistry and Biomedicine, School of Life Sciences, University of Sussex, Brighton, Falmer BN1 9QG, UK; 2 LipidArt Research and Development Ltd, Szeged, Temesvári Street 62, H-6726, Hungary

**Keywords:** Hsp90, FKBP51, Alzheimer’s disease, Molecular chaperone, Proteostasis, LA1011

## Abstract

With increasing age comes the inevitable decline in proteostasis, where chaperone and co-chaperone activity becomes imbalanced. These changes lead to global disturbances and pathogenic rewiring of the chaperone system into epichaperones consisting of protein networks that are ultimately dysfunctional. Such imbalances in proteostasis may favor mechanisms that can lead to neurological diseases, such as Alzheimer’s disease (AD). Consequently, there has been an increase in research activity toward finding small molecules that can re-balance the chaperone and co-chaperone machinery to counter the effects of disease resulting from old age. The Hsp90 co-chaperone FKBP51 has recently been identified as a protein whose induction not only increases with age but is elevated further in AD cells. Significantly, FKBP51 plays a role in the Hsp90-dependent isomerization of tau, which in turn influences its phosphorylation and susceptibility to aggregation. We hypothesize that FKBP51 is a major player that is able to elicit tauopathy in response to amyloid-beta senile plaques that damage the brain. We propose that elevated FKBP51 levels result in an abnormal FKBP51–Hsp90 activity that alters the normal processing of tau, which manifests as hyperphosphorylation and oligomerization of tau. Thus, the Hsp90–FKBP51 complex is emerging as a drug target against AD. In support of this idea, the structure of the FKBP51–Hsp90 complex was recently described, and significantly, the small-molecule dihydropyridine LA1011 was shown to be able to disrupt the Hsp90–FKBP51 complex. LA1011 was previously shown to effectively prevent neurodegeneration in the APPxPS1 AD transgenic mouse model. This review looks at the role of Hsp90 and its co-chaperones in AD with a focus on FKBP51.

## Highlights

• LA1011 was identified as a potential therapeutic against Alzheimer’s disease (AD).
• The molecular target of LA1011 was identified as the C-terminal domain of Hsp90.
• LA1011 disrupts the Hsp90–FKBP51 complex and downregulates tau hyperphosphorylation.
• The Hsp90–FKBP51 complex is a target for small-molecule therapies against AD.

## Graphical abstract

**Figure:**
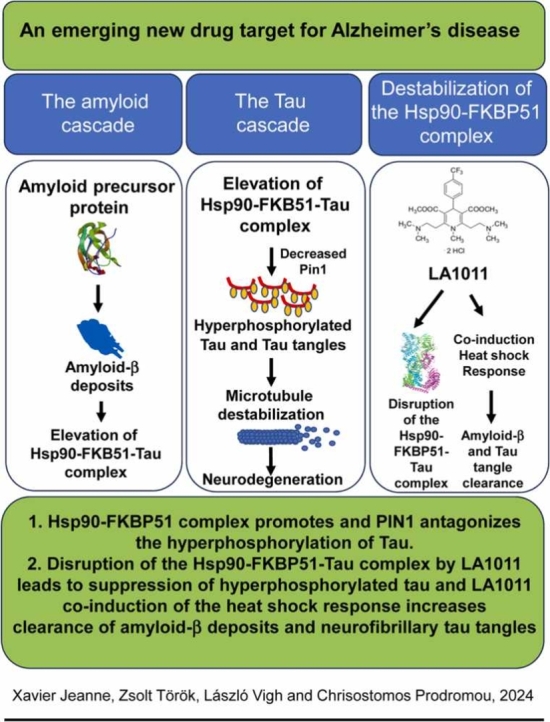

## Introduction

Alzheimer’s disease (AD) is a devastating neurodegenerative disorder that stands as the major cause of dementia and is typically diagnosed as a slowly progressive disease that results in cognitive decline. To date, approximately 50 million people globally are diagnosed with dementia, which consumes 1% of the global economy, although AD is projected to increase to 150 million people by 2050.^1^ Late-onset AD is typically an age-related disease with 95% of cases occurring in adults over the age of 65, while the remaining 5% appear to occur in patients in mid-life.^2^ Typically, late-onset disease is a consequence of age-related changes in combination with a variety of associated risk factors, whereas early onset disease is normally due to genetic mutations that are associated with AD.^3^, ^4^ Rewiring of the chaperone system into epichapromes, which consist of the major heat shock proteins such as Hsp90 together with scaffolding and regulatory proteins, is able to assist cell survival under the elevated proteome demand, which is thought to propagate the progression of AD.^5^, ^6^, ^7^, ^8^, ^9^

Intensive research over many decades has failed to produce an adequate and effective treatment for all AD patients. A variety of registered drugs have collectively failed to display sufficient efficacy.^10^ A new approach involves the use of monoclonal antibodies that target amyloid beta (Αβ) deposits. Aducanumab (marketed as Aduheim) was the first treatment shown to suppress cognitive and functional decline in AD patients by removing Αβ deposits.^11^ It was controversially approved as a treatment for AD by the U.S. Food and Drug Administration in 2021. However, it was later discontinued by its manufacturer (Biogen) in 2024, considering a lack of adequate evidence for clinical efficacy, safety issues, and cost.^12^ However, Lacanemab, another monoclonal antibody targeting Αβ deposits, was approved in July 2023 by the U.S. Food and Drug Administration.^13^, ^14^, ^15^ Lecanemab, also known as BAN2401, at 10 mg/kg biweekly, showed a significant effect on cognitive outcomes in AD.^16^ However, a need for vigilant safety monitoring in clinical practice was acknowledged where a balanced assessment of benefits and potential risks associated with Lecanemab is necessary. Another study showed that, in a sample of 70-year-old patients, only 10.3% were eligible for treatment, of whom only 6.2% did not present factors requiring careful consideration.^17^ In particular, amyloid-related imaging abnormalities such as cerebral microhemorrhages remain a real concern.^18^

Another study, which shows promise, aims to target the epichaperomes network by targeting either Hsp90-incorporating epichapromes with PH-H71 (Zelavespib) or PU-AD (Icapamespib) or GRP94-incorporating epichapromes with PU-WS13.^7^, ^8^, ^19^ Currently, zelavespib and icapamespib have translated to clinic not only in AD but in cancer.^8^, ^20^, ^21^ To date, studies on the safety, tolerability and pharmacokinetics of Icapamespib on healthy nonelderly and elderly subjects support a phase 2 advancement of icapamespib for the evaluation in AD and other neurodegenerative diseases.^22^

**Fig. 1:**
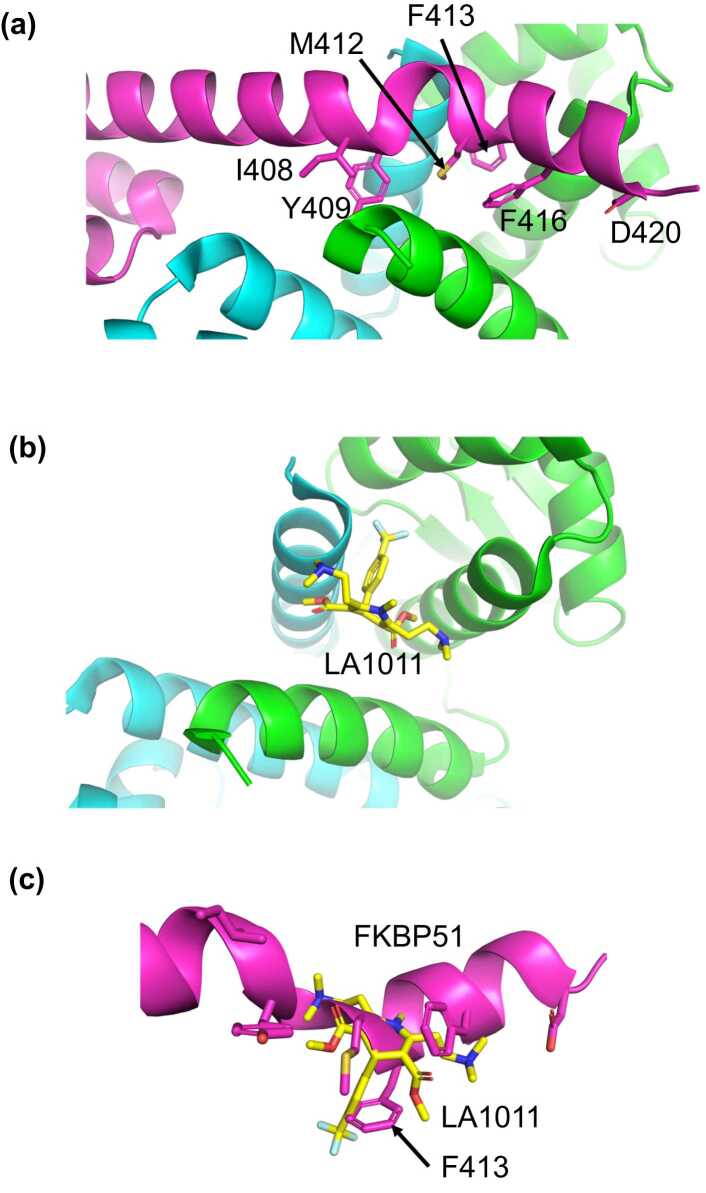
The interaction of Hsp90 with FKBP51 and LA1011. (a) PyMol cartoon of the Hsp90–FKBP51 complex showing the interaction of helix 7 of the TPR domain of FKBP51 (magenta) binding to the C-terminal hydrophobic cleft of Hsp90 (green and cyan). (b) PyMol cartoon of the Hsp90 (green and cyan) in complex with LA1011 (yellow sticks). (c) PyMol cartoon showing the superimposition Hsp90 structures (Hsp90 omitted for clarity) showing a steric clash between the TPR helix of FKBP51 (magenta) and LA1011 (yellow sticks).

However, it is clear we are still in need of an effective treatment for all AD patients, and this review will look at how age-related chaperone imbalances in the human brain offer new emerging drug targets that may provide some hope for patients with this debilitating disease.^23^, ^24^ One such target that is gaining traction is the Hsp90–FKBP51 complex (Figure 1(a)–(c)).^23^

## Amyloid cascade hypothesis

According to the amyloid cascade hypothesis, AD follows a pathological sequence of events that starts with Αβ peptide accumulation, aggregation, and amyloid formation extracellularly within the brain.^25^, ^26^, ^27^ This subsequently elicits a tauopathy in which hyperphosphorylated tau oligomerizes and then aggregates as neurofibrillary tangles (NFT) within neurones, which eventually causes neurological disease. Αβ peptide is 38 to 43 amino acid residues in length formed *via* the sequential cleavage, by the β-secretase and γ-secretase enzyme activities, of the amyloid precursor protein (APP), an integral membrane-bound protein. It is believed that APP functions at the cell surface and has been implicated as a regulator of synapse formation,^28^ neural plasticity,^29^ antimicrobial activity,^30^ and iron export.^31^ Specific forms of Αβ peptide, such as Aβ 1–42 and Aβ 3–40, appear to be more amyloidogenic than others.^32^ It is generally assumed that an imbalance in pathways that utilize the Αβ peptide^33^ or as a consequence of the disruption of homeostatic processes that regulate the proteolytic cleavage of the APP^34^ causes a reduction in Aβ clearance from the brain. This leads to the extracellular accumulation of Aβ peptides that then form oligomers and senile plaques.^34^ In turn, this appears to elicit a tauopathy in which we see a neurotoxic cascade involving membrane damage, mitochondrial dysfunction, cytoskeletal changes, and ultimately, the hyperphosphorylation of tau, which leads to microtubule destabilization^35^, ^36^, ^37^, ^38^ and tau NFT. These changes appear to ultimately cause neuronal dysfunction and cellular death.^39^, ^40^, ^41^, ^42^, ^43^

In the AD brain, the phosphorylation of APP is increased at the Thr 668-Pro motif, which has been proposed to dictate the fate of APP processing, such that a *trans* configuration promotes a nonamylogenic pathway, whereas the *cis* conformation increases the amylogenic pathway.^44^ It has been reported that the PPIase PIN1 binds to the pThr 668-Pro motif, isomerizing proline within full-length APP (or alternatively the C99 fragment of APP^45^), leading to a reduction of Aβ production.^44^ Furthermore, it has been noted that PIN1 overexpression is able to promote nonamylogenic APP processing, whereas a PIN1 knockdown either alone or with an APP mutant causes an age-dependent increase in Aβ 1–42 peptide.^44^ In fact, APP appears to adopt a *trans*-conformation but tends to shift toward a *cis*-conformation following phosphorylation due to local structural constraints.^46^ Since PIN1 (and its homologs) are the only PPIase enzymes known to isomerize proteins in a phosphorylation-dependent manner, it is clear that PIN1 must play a critical role in avoiding the amylogenic pathway for APP^47^, ^48^, ^49^ and is therefore important in regulating protein function.^49^, ^50^, ^51^, ^52^ The role of PIN1 cannot, therefore, be underestimated since proline-directed kinases and phosphatases are conformation-specific and act only on the *trans* conformation, and PIN1 counter acts specific unfavorable *cis* conformations.^53^, ^54^, ^55^, ^56^ Furthermore, the isomerization of Ser/Thr-Pro bonds is further slowed after phosphorylation and renders them resistant to conventional PPIase activity.^49^ It is significant that, during the development of AD, PIN1 function is downregulated and/or inhibited,^27^, ^57^, ^58^, ^59^, ^60^, ^61^ leading to elevated levels of the *cis* pThr 668-Pro motif,^44^ and it has been shown that deletion of PIN1 in mice causes tau and Aβ-related pathologies and neurodegeneration in an age-related manner.^44^, ^62^

During AD onset, tau Thr 231 and Ser 262 appear to be phosphorylated early on,^63^, ^64^, ^65^ and phosphorylation of Thr 231 leads to the phosphorylation of Ser 199, Ser 396, Ser 400, and Ser 404,^66^ which manifests in the hyperphosphorylation of tau.^63^ It appears that phosphorylation of Thr 231 allows kinases such as Gsk3β to access tau and cause further phosphorylation.^66^ However, pThr 231 (but not pSer 262) is a target of PIN1 activity, which can catalyze the isomeric change of the Tau pThr-Pro motif from *cis* to *trans*,^50^, ^67^ thus facilitating its dephosphorylation by the *trans*-specific protein phosphatase 2A (PP2A).^55^, ^68^, ^69^ Consequently, this then appears to cause dephosphorylation at Ser 199, Ser 396, Ser 400, and Ser 404, as observed in rat cultured hippocampal cells under cytotoxic stress conditions induced by treatment with Aβ 1–42 oligomers (not fibrillar aggregates).^70^ Therefore, it appears that PP2A activity antagonizes Gsk3β-dependent phosphorylation of tau,^55^ and that during Aβ treatment, an initial and transient imbalance between Gsk3β and PP2A activity occurs, which favors the latter. It is also significant to note that a PIN1 knockout causes accumulation of the pThr 231-Pro motif in the tangle-specific *cis*-conformation of tau. Thus, it appears that PIN1, by catalyzing a *cis* to *trans* of isomerization of the pThr 231-Pro motif, promotes tau dephosphorylation (by PP2A), which then restores tau function. Thus, this implies that if PIN1 activity drops below a specific threshold, the accumulation of the *cis* pThr231-Pro motif leads to tau hyperphosphorylation, aggregation, and NFT formation.

## The role of Hsp90 and its co-chaperones in Aβ regulation

The accumulation of Aβ is considered a significant contributor to AD.^71^ Although approximately 14% of patients with mild to moderate AD either lack or show very sparse Aβ deposits.^72^ However, it is clear that Aβ deposits are associated with damage to brain tissue, including DNA double-strand breaks^73^, ^74^, ^75^ and their apparent ability to elicit tauopathy.^76^ Molecular chaperones maintain proteostasis, and as such, they are critical regulators of proteins associated with neurodegenerative disease. Consequently, it is no surprise that, from the moment of their production, proteins such as APP, members of the γ-secretase complex, and tau (including a number of neuroinflammatory components) are all in contact with chaperones.^77^ A complete description of the balance of production and clearance of Aβ peptides is key to understanding amyloid plaque proteostasis. The significance of deranged expression of HSPs and various chaperones in AD cases has been reported,^78^, ^79^, ^80^ and it has been shown that 17-AAG, an Hsp90 ATPase inhibitor, can attenuate Aβ toxicity and prevent memory loss in the Tg2576 transgenic AD mice model.^81^ In this mouse model, inhibition of Hsp90 improved synaptic markers and density, *in vivo* long-term potentiation, and memory loss.^81^, ^82^ It appears this effect was mediated by activation of HSF1, which in turn upregulated synaptic genes. It was suggested that HSF1, APP, and Aβ peptide may form a self-regulating system that counters the deleterious effects of Aβ on synaptic function.^83^ Furthermore, most recently, the small-molecule allosteric activator of the ATPase activity of Hsp90, the dihydropyridine LA1011, was shown to decrease Aβ plaque, and tau aggregates in the APPxPS1 AD mice model.^23^, ^84^, ^85^ Furthermore, it should be noted that, although PIN1 does not itself appear to be an Hsp90-dependent client protein, its catalytic activity is modulated by phosphorylation at Ser 16, thus preventing it from interacting with its target motif,^86^ and significantly, it appears that the Hsp90-dependent kinase, protein kinase A, is responsible for inactivating PIN1.^86^, ^87^, ^88^, ^89^ Thus, a decrease in Hsp90 activity could impact indirectly on the isomerase activity of PIN1 by preventing its inhibition, which would favor dephosphorylation at the pSer 262-Pro motif and downregulation of tau phosphorylation as a whole.

A major player that affects Aβ levels is the senile plaque-associated microglia in the brain. HSP-induced microglial activation appears to induce cytokine production ((Interleukin-6) IL-6 and (Tumor necrosis factor alpha)TNF-α), thus providing neuroprotection by increasing phagocytosis and clearance of Aβ peptides.^90^, ^91^ The mechanism by which microglial activation occurs by exogenous HSPs is thought to involve NF-kB and p38 mitogen-activated protein kinase pathways mediated by Toll-like receptor 4 activation. This perhaps offers another opportunity to intervene against AD.

Hsp90 also forms co-chaperone macromolecular complexes that regulate Aβ peptide processing and tau metabolism directly, which ultimately maintain proteostasis.^83^ Pharmacological inhibition of Hsp90 appears to significantly decrease the intracellular levels of phosphorylated tau species in a CHIP-directed and proteasome-dependent manner,^77^, ^92^ which ultimately prevents Aβ-induced neurotoxicity.^93^ The recruitment of CHIP, a Hsp90 co-chaperone with E3 activity, promotes the ubiquitination of tau protein and activates its downstream degradation processes. In *Caenorhabditis elegans*, Hsp40, Hsc70, Hsp90, and STI1 appear to buffer Aβ toxicity, and aged-tissue or within AD diseased-tissue, these chaperone networks are weakened,^94^ which would disturb proteostasis. It has also been reported that the interaction between STI1 and cellular prion protein (PrP^C^) prevents soluble oligomers of Aβ peptide (AβO) from binding to PrP^C^ and that this appears to protect neurons from AβO-induced cell death.^95^, ^96^, ^97^, ^98^, ^99^, ^100^, ^101^, ^102^, ^103^, ^104^, ^105^, ^106^ Hsp90 has also been reported to inhibit the early stages of Aβ aggregation, while the Hsp90–Hsp70–Hsp40 complex appears to inhibit Aβ fibril formation.^107^

Clearly, the interplay of the many systems that regulate the proteostasis of APP and Aβ peptide is complex, and chaperones play an important role. However, while AD is characterized by an abnormal increase of Aβ peptide, it may not in itself be the direct cause of AD.^90^ As such, there may be limited intervention possible once Aβ deposits have been substantially established and other physiological changes in the brain have taken place. Significantly, one such physiological change in response to Aβ deposition is processes that lead to tau pathology, but the mechanism by which Aβ plaques trigger this remains elusive. It is thought that tau may be in a feedback loop with Aβ in AD, where an increase in Aβ accumulation increases NFT formation of tau, which further stimulates Aβ aggregation.^108^, ^109^ Therefore, understanding the mechanism(s) by which Aβ accumulation elicits a tauopathy is crucial in defining how AD manifests itself, and it is no surprise that a complete description of Hsp90’s role in tau phosphorylation is therefore essential.

## The role of Hsp90 and its co-chaperones in tau regulation and NFT formation

Aβ peptide deposits appear to trigger the Hsp90-directed hyperphosphorylation of tau,^110^, ^111^ which subsequently leads to NFT and neurotoxicity.^25^, ^26^, ^27^, ^60^, ^112^, ^113^, ^114^, ^115^, ^116^ However, despite significant amounts of research, further clarification of this pathogenetic mechanism is needed. Furthermore, variability in the onset of AD, as well as other tauopathies, suggests that environmental and genetic factors^117^, ^118^ and the role of chaperone systems and their decline in maintaining proteostasis within aged-tissue or AD-tissue may all be significant factors in generating pathogenic tau species and increasing susceptibility to AD.^119^ Therefore, it is no surprise that changes in the ability of chaperone systems to maintain the correct balance between protein folding and clearance of aberrant proteins^120^ are now gaining traction as a mechanism leading to AD.

With age, it has been reported that transcripts representing Hsp90 and various co-chaperone proteins are altered, such that there is a repression of Hsp90β, Hsp90α, Cyp40, FKBP52, DNAJC7, PP5, TOM70, SGTB, Tah1 (RPAP3), and CDC37, a slight repression of HOP (STIP1), CRN, Aha1, XAP2 (AIP), and p23, while FKBP51, UNC-45A, NASP, CNS1, and DYX1C1 are either induced or slightly induced, and finally CHIP expression remains unchanged.^42^, ^94^, ^121^, ^122^, ^123^, ^124^ However, while these age-related changes undoubtedly affect the ability to maintain proteostasis, diseased cells may show changes in chaperone or co-chaperone levels that are different from those in aged cells, which may further imbalance proteostasis. For example, a number of co-chaperones are repressed in AD cells to levels below those seen in aged cells and include WISp39 and CDC37, although CDC37 has also been reported to be elevated in the AD brain at similar levels seen due to an age-related increase.^111^ In contrast, induction levels of UNC-45A, CHIP, and p23 remain unchanged, while levels of NASP, Aha1, and FKBP51 are induced.^42^, ^121^, ^125^ Whether the differences in chaperone and co-chaperone protein levels seen between AD and nondiseased cells play a role in promoting disease or whether these are changes in response to disease remains an open question.

A number of Hsp90 co-chaperones play a direct role in tau regulation. An important player is CHIP, an E3 ligase co-chaperone of Hsp90 that can drive the ubiquitination and subsequent proteasomal degradation of tau.^126^, ^127^ However, induction levels of CHIP are unchanged in both aged and AD-diseased tissue,^42^, ^94^ suggesting that CHIP activity itself cannot be a direct factor in altering the degradation of tau.

PP5 is a Ser/Thr phosphatase that plays a role in the phosphorylation state of tau, Significantly, PP5 is activated when bound to Hsp90^128^ and can dephosphorylate tau at several phosphorylation sites connected to AD pathology.^129^ Significantly, PP5 is repressed in AD,^42^, ^130^ which may decrease dephosphorylation of tau, which would perhaps favor tau aggregation.

Another important player in tau regulation is CDC37. CDC37 is a co-chaperone of Hsp90 known for its adapter-like function in the assembly of protein kinase–CDC37–Hsp90 complexes. However, CDC37 also plays two important roles in regulating both normal and abnormal tau. Firstly, it appears that CDC37 can directly interact with tau and form tau–CDC37–Hsp90 complex,^111^ and it appears that phosphorylated tau is the preferential client of the CDC37–Hsp90 complex.^92^ Consequently, CDC37 overexpression is able to preserve tau while its suppression destabilizes it.^111^ Inhibition of Hsp90 elicits tau clearance, and the simultaneous suppression of CDC37 potentiates the destabilization of tau. It appears that the knockdown of CDC37 causes a greater reduction of phosphorylated-tau relative to total tau levels. In contrast, CDC37 overexpression, following Hsp90 inhibition, prevented tau degradation. Clearly, the CDC37–Hsp90 complex is responsible for stabilizing tau,^111^ and elevated levels of CDC37 in AD tissue might be a significant factor toward AD. The second means by which CDC37 regulates tau is through its central role in protein-kinase maturation or stabilization of specific protein kinases. Consequently, CDC37 knockdown alters the phosphorylation profile of tau, due to decreased stability of the protein-kinase CDK5 and AKT, while GSK3beta and MARK2 levels were unaffected. Thus, where it has been reported that the cellular levels of CDC37 appear to increase with age, this suggests that an imbalance between clearance and preservation of tau may occur that favors tau stability, which could lead to a tauopathy in time.^111^ However, because it was also reported that CDC37 induction levels remain unchanged with age and are repressed in the AD brain,^42^, ^94^ the evaluation of the role of CDC37 in AD is difficult. However, in contrast to CDC37, the highly homologous 35-kDa protein CDC37L1, which has a 31% similarity to CDC37, appears to have an opposite effect on tau stability.^131^ Hence, overexpression of CDC37L1 caused a decrease in tau, while its knockdown stabilized tau. Clearly, the role of CDC37 and CDC37L1 in tauopathies requires more attention.

Another co-chaperone that stabilizes tau is p23,^132^ and knockdown of p23 results in a reduction of both phosphorylated tau and total tau cellular levels. It appears that p23 can directly interact with tau, in a similar way to its interaction within the Hsp90–HOP–Hsp70–p23–tau complex.^132^ Significantly, the addition of p23 to the Hsp70–HOP–Hsp90 complex slightly delayed modified tau fibrilization and overall reduced it significantly. Thus, as with steroid hormone receptors, the Hsp90–HOP–Hsp70–p23 complex is involved in the stabilization of the tau client protein complex of Hsp90.^133^ Interestingly, *in vitro*, Hsp90 and Hsp70 also delay tau aggregation, but with CDK2-modified tau, which produces a tau phosphorylation profile similar to that seen in AD and has a decreased affinity for Hsp90, whereas Hsp90 alone accelerated modified tau fibrilization.^132^ However, the expression of p23 in AD tissue remains unchanged relative to aged tissue, where it slightly decreases.^42^, ^94^ It was also noted that higher concentrations of CHIP were required to dissociate the Hsp70–HOP–Hsp90–tau–p23 complex relative to the complex lacking p23. The role of p23 might suggest that p23 delays tau degradation but is unlikely to be an important factor in generating tauopathy.

Aha1 was identified as an *a*ctivator of *H*sp90 *A*TPase activity and as such affects the dwell time of the Hsp90 chaperone cycle and therefore the chaperoning of client proteins.^134^, ^135^ Aha1 is known to localize with tau NFT in human AD tissue.^136^ *In vitro*, a combination of Hsp90 and Aha1 dramatically induces tau fibrillation in the presence of ATP, and it was noted that such tau aggregation was greater than for a Hsp90–FKBP51 combination. This suggests the release of tau that is bound to Hsp90 in a form that is prone to fibrillation. Interestingly, the Aha1-binding inhibitor KU-177 dramatically decreased tau aggregation *in vitro*. *In vivo*, the overexpression of Aha1 in the rTG4510 tau transgenic AD mouse model dramatically increases aggregated tau, leading to both neuronal loss and cognitive impairment.^136^ A similar study also showed that overexpression of Aha1 increased pathological tau levels in 16-month-old wild-type mice and impaired associative learning.^137^ Furthermore, similar studies with a C-terminal-binding inhibitor, SWE84, of Aha1 preferentially cleared phosphorylated and aggregated prone tau.^138^ It may be significant that Aha1 levels are induced in AD brain tissue while they are slightly repressed in aged tissue.^94^, ^136^

Hsp90 also associates with the peptidylprolyl isomerase (PPIase) immunophilins, FK506-binding protein 51 (FKBP51), and FKBP52 and with cyclophilin 40 (Cyp40, also known as peptidylprolyl isomerase D).^42^ Cyp40 is thought to provide a neuroprotective function since it was recently shown to disaggregate tau fibrils *in vitro* and, significantly, to prevent toxic tau accumulation *in vivo.*^139^ Unfortunately, Cyp40 expression levels not only decrease with age; they are repressed in AD.^42^, ^94^

In contrast, FKBP52 induces tau aggregation by a mechanism that involves a direct molecular interaction with truncated and wild-type tau.^140^, ^141^ Elevated levels of FKBP52 have been shown to increase pathological tau in 16-month-old wild-type mice, to cause defects in cognitive flexibility during spatial learning, and to evoke a neuroinflammatory response.^137^ Fortunately, FKBP52 levels appear to be lower in the cortex of AD patient brains, and it appears that FKBP52 is not a major driver of tau pathology in this case.^94^, ^122^

Unlike FKBP52, FKBP51 levels not only progressively increase with age but further increases are seen in AD patients.^42^, ^94^, ^121^, ^125^ Stress, which is also associated with AD, is also known to increase FKBP51 levels.^142^, ^143^ FKBP51 was shown to be able to preserve toxic tau oligomers *in vivo*^25^ and decreased tau levels are found in the brain of mice lacking FKBP51.^110^, ^121^ Elevated FKBP51 levels, therefore, seem to be a prime factor that may be able to elicit a tauopathy. Far less is known with regards to other FKBPs (FKBP12, 22, 25, 37, 38, etc), cyclophilins (such as CypA, B, and 40 etc), and PPIase-like proteins (such as PPIase-like [PPIL]1, Cyp-like 1, [CypL1], PPIL2 [Cyp60], PPIL3 [CypJ], PPIL4, PPIL5, and PPIL6) and their role in AD.^144^

Finally, S100A1 has been identified in complex with Hsp90, Hsp70, FKBP52, Cyp40,^145^ and STIP1.^146^ S100A1 can cause a ca^2+^-dependent microtubule disassembly in glioma cells and myoblasts, which suggests a possible role in tau pathology.^147^

## FKBP51 is a prime suspect for driving tau pathology in AD

While other co-chaperones of Hsp90 might affect tau phosphorylation and stability, it is clear that our current understanding is that FKBP51, FKBP52, CYP40, PP5, CDC37, Aha1, and CHIP appear to be major players. Consequently, any imbalance in their cellular levels may have major implications for the development of AD.^42^ However, only FKBP51, CDC37, and Aha1, which are able to either stabilize tau or promote tau aggregation, are also elevated in AD tissue.^94^

The structure of the FKBP51–Hsp90 complex was recently reported, and it shows that the C-terminal or seventh helix of the (Tetratricopeptide) TPR domain of FKBP51 is bound across a hydrophobic cleft between the dimer interface at the extreme end of the C-terminal domain of Hsp90 (PDB 7L7I and 8FFW) (Figure 1(a)–(c)).^148^, ^149^ Similarly, the structure of Hsp90–tau complex has been described^150^ The interaction of FKBP51 with Hsp90 is critical for aligning the active PPIase domain of FKBP51 with the bound tau client, and failure to correctly interact would influence proline *cis*–*trans* isomerization of tau that in turn would alter the phosphorylation of tau itself.^56^, ^70^, ^151^, ^152^ Specifically, FKBP51 appears to promote the isomerization of Pro 231 and Pro 233, critical residues for tau's conformation and function. The isomerization activity of FKBP51 at Pro 231 and Pro 233 appears to then alter the phosphorylation pattern of tau and encourages the formation of tau oligomers, which ultimately form NFT.^121^ Consequently, it is interesting to speculate whether an overactive FKBP51 activity might overcome the beneficial activity of PIN1, pushing tau toward hyperphosphorylation, oligomerization, and finally aggregation, especially as PIN1 expression levels decrease in AD.

**Fig. 2:**
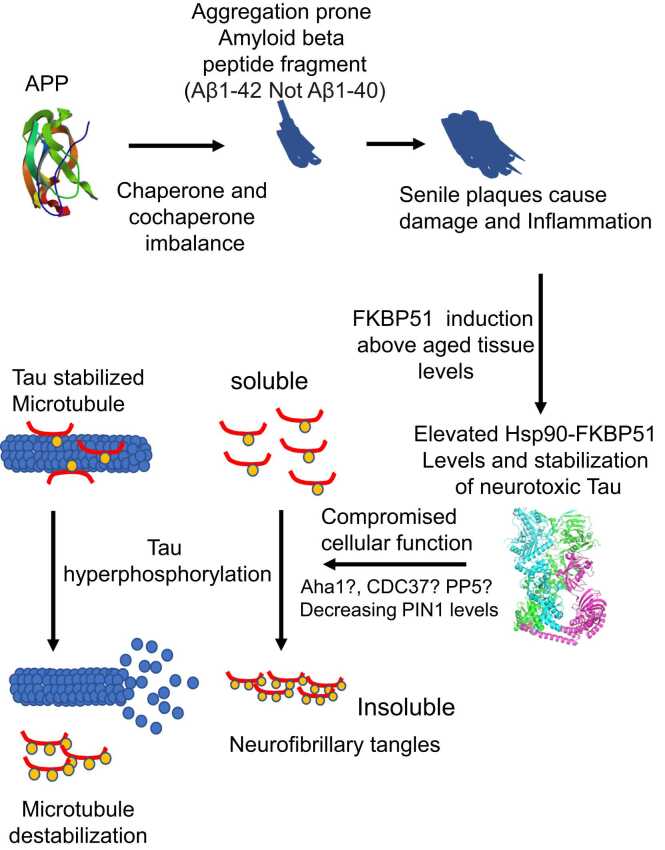
Proposed events in the development of AD. An imbalance of chaperones and co-chaperones occurring with age leads to changes in clearance of Aβ peptide that aggregate to form senile plaques. Damage and inflammation might induce FKBP51 that impacts on the normal regulation of tau phosphorylation. In combination with other co-chaperone changes or in complex with Hsp90 alone, FKBP51 drives the hyperphosphorylation of tau, which leads to NFT formation and destabilization of microtubules as part of the tauopathy. Abbreviations used: Aβ, amyloid beta; AD, Alzheimer’s disease; APP, amyloid precursor protein; NFT, neurofibrillary tangles.

We hypothesize that since FKBP51 is a prime factor that is upregulated during a stress response, it is conceivable that Aβ deposits elicit upregulation of FKBP51 due to the damage and inflation they may cause in the AD brain. This, in turn, affects the *cis*–*trans* isomerization and consequently the phosphorylation of tau,^121^, ^152^, ^153^ which may be further influenced negatively by other co-chaperones such as CDC37 and Aha1, which ultimately leads to tau fibrillation. Furthermore, the activity of PIN1 may be insufficient to counter increases in the *cis* isomerization of tau, especially as PIN1 levels and activity decline as AD progresses. Consequently, FKBP51 may represent the major factor that elicits tau pathology when specific conditions are met by destabilizing the normal regulation of tau phosphorylation due to its elevated levels. FKBP51 would, therefore, represent the elusive link between Aβ deposits and the drive toward the gradual formation of tau NFT and neurological disease (Figure 2).

Recognizing the role of FKBP51 in driving the formation of NFT allows for the intervention of effective treatment methods. Potential drugs could either reduce tau levels or inhibit tau accumulation and aggregation. Consequently, there is a drive toward developing small molecules that can restore age-related decline in proteostasis or to develop Hsp90 inhibitors that promote the degradation of tau.^92^, ^154^, ^155^

Toward restoring an age-related decline in proteostasis, the dihydropyridine, LA1011, was selected on the basis of its Hsp co-inducing effect (enhanced induction above an already induced state) and its favorable toxicological data.^84^ Remarkably, a 6-month treatment plan of LA1011 administration improved the spatial learning and memory functions in wild-type mice and eliminated neurodegeneration in the APPxPS1 double mutant mouse model. Significantly, the Hsp co-inducer effect on the transgenic AD mice leads to favorable changes, including preserving the number of neurons, increasing dendritic spine density, and reducing tau pathology and amyloid plaque formation. Clearly, these results suggest that LA1011 has potential as a pharmaceutical candidate that can be developed for the therapy of neurodegenerative diseases, particularly AD. Significantly, soon after these observations, LA1011 was shown to target the extreme C-terminal domain of Hsp90 and to activate its ATPase activity,^85^ and a co-crystal structure of LA1011 and Hsp90 has now opened the doorway to a structure-based driven strategy for developing LA1011.^23^

The positive effects on AD mouse models seen with LA1011 treatment and the ability of LA1011 to prevent FKBP51 interaction with Hsp90 corroborate the importance of FKBP51 in driving tauopathies.^23^ It appears that LA1011 directly competes with the binding of the terminal helix of the TPR domain of FKBP51 (Figure 1(a)–(c)), thus disrupting its ability to catalyze proline isomerization of tau, which ultimately decreases phosphorylation events that lead to tau NFT and neurotoxicity. There is now a growing amount of evidence that supports this hypothesis.^144^, ^156^ Consequently, it is not surprising that there is a push to develop LA1011 as a clinical candidate against AD. Alternatively, small molecules that inhibit FKBP51 directly, either inhibiting its PPIase activity or blocking its TPR domain form binding to Hsp90, could be developed.^157^ It is noteworthy that there is evidence that FK506 has been shown to reduce Aβ and tau levels in the hippocampus and cortex of 3xTg-AD mice.^158^ Unfortunately, FK506 displays adverse side effects.^159^

## Concluding remarks and future directions

There is growing evidence that AD is a result of an imbalance in the homeostatic processes that regulate APP, Aβ peptides, and tau.^33^, ^34^, ^119^, ^120^ Hsp90 is emerging as a central regulator of tau pathology, and many of its co-chaperones influence or regulate tau proteostasis. The most important appears to be FKBP51, an immunophilin that is known to stabilize tau and whose expression is known to increase, not only due to stress or damage of brain tissue but a factor known to be elevated in AD tissue.^42^ Recent work has shown that a small molecule, a dihydropyridine known as LA1011, was able to eliminate neurodegeneration in the APPxPS1 double mutant AD mouse model.^84^ LA1011 was shown to be an activator of the Hsp90 ATPase activity and also a competitive inhibitor against FKBP51 binding to Hsp90.^23^, ^85^ It appears that LA1011 may be able to restore the homeostatic mechanism of the Hsp90–FKBP51 complex such that tau phosphorylation returns toward a normal balanced level, thus avoiding tau hyperphosphorylation. Furthermore, LA1011 causes co-induction of the heat shock response, representing an additional elevation in chaperone and co-chaperone levels above those normally found in AD-diseased tissue.^84^ The additional boost in chaperone and co-chaperone levels appears to enhance the clearance of Aβ and NFT in the APPxPS1 double mutant AD mouse model,^84^ thus downregulating the stimulating factors (protein deposits) driving tauopathy. The importance of elevated CDC37 and Aha1 and decreased levels of PP5 in AD tissue and their interplay with FKBP51 remains to be seen, but encouraged by our initial findings, we are now actively developing LA1011 and evaluating derivatives toward developing a clinical trial candidate against AD. However, although we are optimistic, we remain cautious because many co-chaperones and clients interact with Hsp90, and there is uncertainty as to how the processes they regulate could be affected and what adverse side effects could arise. Consequently, we are currently evaluating the effect of LA1011 on a variety of Hsp90 complexes.

## Funding and support

CP, LV, ZT, and XJ are grateful to the BBSRC and LipidArt (grant number *BB/T008768/1)* who supported this work.

## Author contributions

**László Vigh:** Writing – review & editing. **Zsolt Török:** Writing – review & editing. **Xavier Jeanne:** Writing – original draft. **Chrisostomos Prodromou:** Writing – original draft.

## Declaration of generative AI in scientific writing

Generative AI was not used in writing this manuscript.

## Declarations of interest

The authors declare that they have interests in developing LA1011 as a therapeutic toward managing Alzheimer’s disease.

## Data Availability

No data were used for the research described in the article.
